# Iteradensovirus from the Monarch Butterfly, *Danaus plexippus plexippus*

**DOI:** 10.1128/genomeA.00321-14

**Published:** 2014-04-17

**Authors:** Qian Yu, Peter Tijssen

**Affiliations:** INRS, Institut Armand-Frappier, Université du Québec, Laval, Quebec, Canada

## Abstract

The 5,006-nucleotide (nt)-long genome of a new virus from monarch butterfly pupae was cloned and sequenced. It was flanked by inverted terminal repeats (ITRs) of 239 nt with 163-nt hairpins. The monosense genome with three open reading frames is typical of the genus *Iteradensovirus* in the subfamily *Densovirinae* of the family *Parvoviridae*.

## GENOME ANNOUNCEMENT

Monarch butterflies (*Danaus plexippus plexippus*) migrate from eastern and central North America for overwintering in Mexico. Migration of this emblematic butterfly has been in rapid decline in recent years, prompting the presidents of the United States and Mexico and the Prime Minister of Canada to discuss this problem during a meeting in February 2014. Several factors may be responsible for this trend. The cool and relatively moist high mountain habitats of Oyamel fir forests are ideal for both the firs and the butterflies. The forest canopy and the clustering of the monarchs protect them against freezing (1). Severe logging and climate change threaten these forests, and a massive reforestation effort is under way to reverse this trend. Second, the extensive use of genetically modified herbicide-resistant soybeans and corn may be reducing the number of larval host plants, milkweeds, especially in their main habitat in the Corn Belt (2–4), encouraging the suggestion of a milkweed corridor. However, this has been disputed elsewhere (5). Third, pathogens such as bacteria, parasites, and viruses may affect monarch populations (6–8).

Virus was partially puriﬁed from three infected pupae obtained from a butterfly farm in Granby (Quebec, Canada) by the method described for *Galleria mellonella* densovirus (9) and visualized by electron microscopy. A preliminary genome characterization was obtained with the sequence-independent single-primer ampliﬁcation (SISPA) method (10–12), showing two SpeI restriction sites in a preliminary 4.7-kb sequence. Viral DNA was then blunt ended by a mixture of Klenow large-fragment and T4 DNA polymerase, digested with SpeI, and cloned into EcoRV and SpeI sites in the pBluescriptSK II(-) vector, yielding clones with 3.4-kb inserts and clones with 1.5-kb inserts. Sequences of several complete clones, obtained in both directions with Sanger’s method (10, 11), were identical except for the ﬂip-ﬂop sequences in the hairpins. The sequence between the two SpeI sites was obtained after PCR amplification with gene-specific primers.

The *D. plexippus plexippus* iteradensovirus (DppIDV) genome contained the typical inverted terminal repeats (ITRs) of members of the *Iteradensovirus* genus (*Bombyx mori* densovirus 1 [BmDNV-1], *Casphalia extranea* densovirus [CeDNV], *Sibine fusca* densovirus [SfDNV], *Papilio polyxenes* densovirus [PpDNV], and *Dendrolimus punctatus* densovirus [DpDNV]) (10, 11, 13–15). The 239-nucleotide (nt) ITRs with 163-nt terminal J-shaped hairpins were about 90% conserved with those of the other iteradensoviruses. The overall sequence was about 86% identical to CeDNV, about 84% identical to SfDNV and BmDNV, about 78% identical to PpDNV, and about 71% identical to DpDNV.

Similar to other iteradensoviruses, the DppIDV monosense genome contained three intronless genes with essentially identical positions and sizes. The largest, open reading frame 1 (ORF1) (nt 360 to 2618), had a coding capacity of 752 amino acids (aa) and the typical nucleoside triphosphatase (NTPase) motif for NS1. ORF2 (nt 2677 to 4710), with the phospholipase A2 motif, typical for parvovirus VP, had a coding capacity of 677 aa. ORF3, with a 451-aa coding capacity (nt 487 to 1842) corresponded to NS2 and overlapped NS1 at its N terminus. As a comparison, NS1 is aa 753 to 775, NS2 is aa 451 to 455, and VP is aa 668 to 681 for the other iteradensoviruses.

### Nucleotide sequence accession number.

The GenBank accession no. for DppIDV is KF963252.
